# Obesity-Driven Metabolic Disorders: The Interplay of Inflammation and Mitochondrial Dysfunction

**DOI:** 10.3390/ijms26199715

**Published:** 2025-10-06

**Authors:** Wooyoung Choi, Gun Ha Woo, Tae-Hwan Kwon, Jae-Han Jeon

**Affiliations:** 1 Department of Biomedical Science, Graduate School, Kyungpook National University, Daegu 41944, Republic of Korea; daniel202@knu.ac.kr (W.C.); ha8022@knu.ac.kr (G.H.W.); 2 Department of Biochemistry and Cell Biology, School of Medicine, Kyungpook National University, Daegu 41944, Republic of Korea; 3 Department of Internal Medicine, School of Medicine, Kyungpook National University, Kyungpook National University Chilgok Hospital, Daegu 41404, Republic of Korea

**Keywords:** obesity, metabolic disorder, mitochondrial dysfunction, inflammation

## Abstract

Obesity contributes to the development of metabolic disorders such as type 2 diabetes mellitus (T2DM) and metabolic dysfunction-associated steatotic liver disease (MASLD) through sustained low-grade inflammation and mitochondrial dysfunction. In obesity, hypertrophied adipose tissue release high levels of pro-inflammatory cytokines, including TNF-α, IL-6, and IL-1β, and elevates circulating free fatty acids. These changes promote systemic insulin resistance and ectopic lipid deposition. Mitochondrial dysfunction, including reduced oxidative phosphorylation, excess reactive oxygen species (ROS) production, and mitochondrial DNA damage, further stimulate inflammatory pathways such as the NLRP3 inflammasome, creating a feedback loop that worsens metabolic stress. Ultimately, this interaction disrupts energy balance, weakens insulin signaling, and accelerates β-cell dysfunction and hepatic steatosis. In both T2DM and MASLD, oxidative stress, defective mitochondrial quality control, and dysregulated immunometabolic responses are consistently observed pathophysiological features. Interventions aimed at reducing inflammation and restoring mitochondrial function—including lifestyle modification, mitochondria-targeted therapies, inflammasome regulation, and enhancement of mitochondrial biogenesis or mitophagy—may retard disease progression.

## 1. Introduction

Obesity is a worldwide epidemic defined by excessive fat accumulation that impairs metabolic homeostasis and increases the risk of multiple diseases [1,2,3]. It is a primary cause of metabolic disorders such as type 2 diabetes, metabolic dysfunction-associated steatotic liver disease (MASLD), cardiovascular diseases, musculoskeletal disorders, respiratory disorders, and several cancers [4,5]. Chronic low-grade inflammation and mitochondrial dysfunction play critical roles in the pathophysiology of obesity-induced metabolic disorders [6,7,8,9]. Obesity-induced adipose tissue dysfunction enhances pro-inflammatory cytokine production, exacerbating systemic inflammation and reducing insulin sensitivity [6,8,10]. Concurrently, mitochondrial dysfunction impairs energy metabolism, raises reactive oxygen species (ROS) levels, and exacerbates oxidative stress [6,7,11,12]. This reciprocal interaction between inflammation and mitochondrial dysfunction prolongs metabolic dysregulation, which contributes to the progression of the disease [7,8,13].

In addition to these processes, other pathogenic pathways are also implicated in obesity-related metabolic disorders. Endoplasmic reticulum (ER) stress activates the unfolded protein responses, which not only disrupts insulin receptor signaling but also aggravates lipid accumulation in the liver [14]. Autophagy defects that are independent of mitochondrial quality control reduce the clearance of protein aggregates and lipids, thereby impairing cellular homeostasis and contributing to dysfunction in multiple tissues [15]. Alterations in the gut microbiome further contribute to metabolic endotoxemia and chronic low-grade inflammation by increasing intestinal permeability and endotoxin exposure [16]. While these mechanisms play critical roles, this review will primarily focus on the reciprocal interactions between inflammation and mitochondrial dysfunction. Among the wide spectrum of obesity-related conditions, we specifically concentrated on type 2 diabetes mellitus (T2DM) and MASLD as representative models, to provide a more focused and coherent discussion.

## 2. Pathophysiological Insights into Obesity-Driven Metabolic Disorder

### 2.1. Type II Diabetes Mellitus (T2DM)

#### 2.1.1. Overview of T2DM

Type 2 diabetes mellitus (T2DM) is a chronic metabolic disorder characterized by persistent hyperglycemia resulting from insulin resistance in peripheral tissues coupled with an inadequate compensatory insulin secretion by pancreatic β-cells [17,18]. This definition distinguishes T2DM from type 1 diabetes mellitus (T1DM), in which autoimmune β-cell destruction causes an absolute insulin deficiency. T2DM accounts for the vast majority of diabetes cases worldwide and has reached epidemic proportions, closely linked to rising obesity prevalence [19,20]. Recent International Diabetes Federation reports estimate ~537 million adults with diabetes in 2021 and project ~783 million by 2045 [21]. In particular, diabetes mellitus (DM) is associated with body fluid and electrolyte imbalances, hypertension, dyslipidemia, and vascular abnormalities; thus it is also a leading cause of end-stage renal disease that requires renal replacement therapy, such as dialysis or transplantation [22,23,24]. This dramatic increase poses enormous public health and socioeconomic challenges.

Excess adiposity is one of the strongest risk factors for T2DM development. The majority of patients with T2DM are overweight or obese, and longitudinal studies confirm that weight gain significantly increases the risk of developing T2DM, whereas weight loss can prevent or delay its onset [25,26]. However, obesity is not a sufficient or universal cause. Importantly, not all obese individuals develop T2DM, and not all T2DM patients are obese [27]. Approximately 10–20% of T2DM cases occur in normal-weight or lean individuals [28]. This phenomenon is often associated with increased visceral fat deposition or other genetic and ethnic susceptibilities. For example, certain Asian populations experience high T2DM rates at relatively lower BMIs. Conversely, around 20–30% of individuals with obesity remain metabolically healthy and free of diabetes [28]. Genetic architecture may partly explain why many East Asians develop T2DM at lower BMI. Common intronic variants at KCNQ1 (e.g., rs2237892/rs2237895/rs2237897) were first identified in Japanese GWAS and replicated across East Asians, associating not only with T2DM but also with impaired fasting glucose and lower HOMA-β, consistent with diminished insulin secretion [29]. Moreover, East-Asian–enriched missense variants in PAX4 (notably p.Arg192His/p.Arg192Ser) alter β-cell development and function and are linked to ear-lier-onset diabetes [30]. These observations underscore that factors beyond generalized obesity—including fat distribution, adipose tissue function, genetics, and even gut microbiome composition—determine metabolic risk.

**Figure 1:**
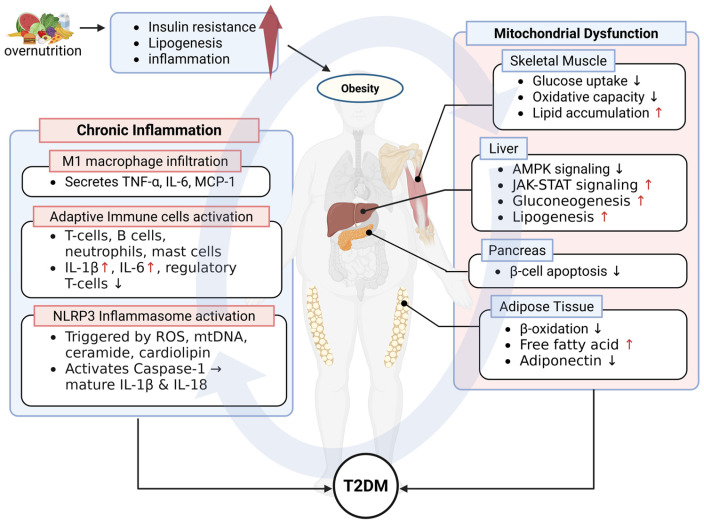
Interplay between mitochondrial dysfunction and chronic inflammation in obesity associated type 2 diabetes mellitus (T2DM). Schematic representation illustrating the mechanistic pathways that connect obesity to T2DM. Excessive nutrition promotes obesity by increasing insulin resistance, lipogenesis, and systemic inflammation. In metabolic tissues, mitochondrial dysfunction is characterized by reduced adenosine triphosphate (ATP) generation, impaired electron transport chain (ETC) activity, elevated reactive oxygen species (ROS) production, and mitochondrial DNA (mtDNA) damage. These changes result in decreased β-oxidation and adiponectin secretion in adipose tissue, reduced glucose uptake and oxidative capacity in skeletal muscle, altered hepatic AMP-activated protein kinase (AMPK) and Janus kinase—signal transducer and activator of transcription (JAK–STAT) signaling, increased gluconeogenesis and lipogenesis, and pancreatic β-cells apoptosis. Damaged mitochondria release ROS and mtDNA, which activate inflammatory pathways such as NOD-like receptor protein 3 (NLRP3) inflammasome and nuclear factor kappa-light-chain-enhancer activated B cells (NF-κB), further amplifying inflammation. Chronic inflammation involves infiltration of M1 macrophages and activation of adaptive immune cells (T helper type 1 [Th1], Th17, B cells, neutrophils, mast cells), secreting pro-inflammatory cytokines including tumor necrosis factor—alpha (TNF-α), interleukin (IL)-6, and IL-1β, which impair insulin signaling and β-cell function. NLRP3 inflammasome activation, triggered by ROS, mtDNA, ceramides, and cardiolipin, activates caspase-1, leading to the maturation of IL-1β and IL-18. Inflammatory cytokines further impair mitochondrial function, creating a self-perpetuating cycle that exacerbates insulin resistance, β-cell dysfunction, and progression to T2DM (Created in https://BioRender.com).

In addition to these genetic contributions, accumulating evidence highlights the roles of epigenetic modifications (e.g., DNA methylation, histone modification) in human obesity and T2DM pathogenesis [31]. Environmental influences, including diet and lifestyle, are also well-established contributors to metabolic risk [32]. These determinants help explain why some obese individuals remain metabolically healthy while others rapidly progress to diabetes. Understanding why only a subset of those with obesity or a genetic predisposition progress to T2DM is an active area of research, highlighting the complexity of T2DM pathogenesis (Figure 1) [33]. For example, KCNQ1 variants impair β-cell repolarization and predispose to mitochondrial stress [34,35], while PAX4 mutations compromise β-cell differentiation and increase inflammatory susceptibility [36]. Importantly, they converge on inflammatory and mitochondrial pathways, thereby linking inter-individual differences to the central mechanistic framework emphasized in this review [37].

#### 2.1.2. Mitochondrial Dysfunction in T2DM Pathogenesis

Mitochondria play a pivotal role in cellular energy homeostasis, oxidizing glucose and fatty acids to generate ATP through oxidative phosphorylation (OXPHOS). Accordingly, mitochondrial impairment is widely observed in T2DM and can disrupt metabolic balance [6,7,37,38]. Numerous studies have linked insulin resistance in T2DM with mitochondrial abnormalities across insulin-sensitive tissues (skeletal muscle, adipose tissue, and liver) and in pancreatic β-cells [5,27,33,37]. Some reports suggest that impaired mitochondrial oxidative capacity and increased mitochondrial oxidant burden precede and contribute to insulin resistance [39]. However, human studies indicate that mitochondrial capacity is not always diminished in insulin resistance: skeletal muscle from obese individuals with and without T2DM showed comparable mitochondrial content and respiration [40], and in athletes with high intramyocellular lipid, oxidative flux remains high despite lipid accumulation (the “athlete’s paradox”) [41]. However, whether these mitochondrial defects are a primary cause of insulin resistance or a consequence of other metabolic disturbances remains debated [42,43]. For instance, some interventions that improve insulin sensitivity—such as exercise training—can do so without markedly increasing mitochondrial content or function, suggesting a more complex, context-dependent relationship between mitochondria and insulin action [44,45]. One key link between obesity-induced metabolic stress and insulin resistance in T2DM is mitochondrial dysfunction: nutrient excess and inflammation can impair mitochondrial oxidative metabolism in muscle and liver, leading to reduced ATP generation, accumulation of reactive oxygen species (ROS), and an inability to switch fuels known as metabolic inflexibility [46,47]. Mitochondrial abnormalities within β-cells further diminish insulin release.

Skeletal muscle, which accounts for the majority of insulin-mediated glucose uptake, often exhibits significant mitochondrial abnormalities in T2DM. Diabetic and insulin-resistant individuals typically show reduced mitochondrial content and oxidative capacity in muscle fibers along with lower expression of electron transport chain components [37,48]. For example, one classic study found that muscle biopsies from insulin-resistant offspring of T2DM patients had ~38% lower mitochondrial density compared with controls [49]. Such deficits in muscle mitochondria lead to decreased fatty acid β-oxidation and ATP production, coupled with excess ROS production [6,11,12,50,51]. This energy shortfall forces muscle cells to accumulate lipid intermediates like diacylglycerols (DAG) and ceramides that can activate stress signaling pathways [10,52,53,54]. In particular, DAG accumulation in muscle can activate novel PKC isoforms (e.g., PKCθ), which then phosphorylate and inhibit insulin receptor signaling, while ceramides may antagonize insulin action by impairing Akt phosphorylation [55,56]. These mechanisms link mitochondrial fuel-handling deficiencies to the development of insulin resistance in muscle.

Adipose tissue mitochondria are also affected in obesity and T2DM. Studies have documented downregulation of PGC-1α and other mitochondrial biogenesis factors in the adipose tissue of obese individuals [6,10,11,53], along with reduced mitochondrial density and fatty-acid oxidation capacity [57]. This mitochondrial insufficiency in white adipose tissue leads to incomplete fat oxidation and enhanced release of free fatty acids (FFAs) into circulation, promoting ectopic fat deposition in the liver and muscle that exacerbates insulin resistance [6,53]. Dysfunctional fat-cell mitochondria may also perturb adipokine secretion (e.g., lowering adiponectin) and create a pro-inflammatory state in adipose tissue, further aggravating systemic insulin resistance [58].

Additionally, obesity is often associated with reduced activity of brown adipose tissue (BAT), whose mitochondria-rich, UCP1-expressing adipocytes normally dissipate energy as heat. A decline in BAT thermogenesis can contribute to a positive energy balance and worsen metabolic health, as active BAT has been shown to improve insulin sensitivity and lipid homeostasis in humans [28].

In the liver, insulin-resistant T2DM patients show mitochondrial changes similar to those seen in metabolic dysfunction-associated steatotic liver disease (MASLD). Liver mitochondria in T2DM have decreased OXPHOS capacity and efficiency, along with increased ROS generation [50,59,60,61]. These impairments promote hepatic fat accumulation (steatosis) and inflammation, which in turn contribute to hepatic insulin resistance.

Pancreatic β-cells, which require robust mitochondrial function for glucose-stimulated insulin secretion (GSIS), are also vulnerable to mitochondrial dysfunction in T2DM. In healthy β-cells, mitochondrial oxidation of glucose generates the ATP needed to close K_ATP channels and trigger insulin release [62,63,64]. Consistent with this, islets isolated from T2DM patients have shown marked alterations in mitochondrial morphology and function. Electron microscopy studies report abnormally swollen or enlarged mitochondria in the β-cells of T2DM patients, accompanied by impaired mitochondrial membrane potential and ATP generation [51,65]. Such defects blunt GSIS and predispose β-cells to failure. Chronic hyperglycemia and elevated FFAs place further stress on β-cell mitochondria: the fuel surplus drives excessive ROS production and destabilizes the mitochondrial membranes. Over time, this leads to the activation of intrinsic apoptotic pathways in β-cells (e.g., via caspase activation), accelerating β-cell apoptosis and loss of insulin-producing mass [66,67,68,69]. The result of this mitochondrial-mediated β-cell attrition is a worsening insulin secretory deficit that, alongside peripheral insulin resistance, propels the progression of diabetes.

Uncontrolled hyperglycemia itself can further damage mitochondria and create a vicious cycle. When blood glucose is persistently elevated, mitochondrial electron transport chains become overloaded with reducing equivalents, leading to excessive leakage of electrons and overproduction of superoxide and other ROS [11,70,71]. The surplus of mitochondrial ROS inflicts oxidative damage on mitochondrial proteins and DNA, impairing the respiratory chain and lowering ATP output in a self-perpetuating manner [11,70,71]. Mitochondrial DNA (mtDNA) is particularly susceptible to mutations and deletions generated by ROS due to its closeness to the electron transport chain (ETC) and absence of histone protection. Accumulation of mtDNA damage may hamper the expression of critical respiratory chain subunits, thereby exacerbating mitochondrial inefficiency. Furthermore, damaged or stressed mitochondria can release mtDNA fragments into the cytosol and even into the bloodstream, where it acts as a “danger signal” or damage-associated molecular pattern (DAMP) [13,71]. This cell-free mtDNA (cf-mtDNA) can engate innate immune sensors—for example, Toll-like receptor 9 in endosomes and the cGAS-STING pathway in the cytosol—thereby activating pro-inflammatory signaling cascades [72]. In this way, mitochondrial dysfunction links to chronic inflammation, as mitochondrial ROS and cf-mtDNA engage the immune system and cytokine production.

Collectively, mitochondrial defects across muscle, adipose, liver, and β-cells create an “energy crisis” and oxidative/inflammatory milieu that undermines insulin action and insulin secretion in T2DM (Figure 1). While it remains unclear whether these mitochondrial abnormalities are a primary cause or a secondary consequence of insulin resistance [42,43], there is no doubt that mitochondrial dysfunction amplifies the metabolic disturbances of diabetes. Therapeutic strategies that bolster mitochondrial function—such as exercise training, which stimulates mitochondrial biogenesis or agents that enhance mitochondrial oxidative efficiency—have demonstrated improvements in insulin sensitivity and glucose homeostasis, underscoring the central role of mitochondrial health in T2DM pathogenesis [73].

#### 2.1.3. The Role of Chronic Inflammation in T2DM

The systemic inflammation associated with obesity aggravates oxidative stress via cytokine-mediated effects on mitochondria, creating a vicious cycle. Emerging research highlights additional mechanistic links, such as adipose tissue-derived exosomal microRNAs that propagate insulin resistance and inflammation to distant organs [74], and the role of the gut microbiome in modulating obesity-related inflammation and metabolic homeostasis [42,75]. In the following sections, we will delve deeper into how these interconnected mechanisms—particularly meta-inflammation and mitochondrial dysfunction—contribute to T2DM and its complications, and how they might be targeted to improve metabolic health.

Pathophysiologically, T2DM is an obesity-driven metabolic disorder where chronic inflammation and metabolic stress work together to trigger and accelerate the disease [46,76]. In states of excess nutrition and adiposity, adipose tissue becomes dysregulated. Adipocytes enlarge (hypertrophy) beyond their healthy storage capacity, leading to localized hypoxia, adipose tissue fibrosis, and the recruitment of immune cells (particularly macrophages) into fat depots [77]. These changes precipitate a shift toward a pro-inflammatory state often termed “metaflammation” [78]. Enlarged, stressed adipocytes and infiltrating immune cells secrete abnormal levels of free fatty acids (FFAs) and adipokines. Circulating FFAs spill over to ectopic sites (such as liver and muscle), where they accumulate as toxic lipid metabolites that impair insulin signaling [25]. Meanwhile, pro-inflammatory adipokines like tumor necrosis factor-α (TNF-α), interleukin-6 (IL-6), and resistin are elevated, while insulin-sensitizing adipokines (e.g., adiponectin) are suppressed [26]. This combination of lipotoxicity and inflammatory signaling induces insulin resistance in peripheral tissues (muscle, liver) and places an increased secretory demand on pancreatic β-cells (Figure 1). Initially, β-cells compensate by hypersecreting insulin. However, chronic metabolic stress (glucotoxicity and lipotoxicity) eventually leads to β-cell dysfunction and apoptotic loss, causing insufficient insulin production. Chronic low-grade inflammation is a key mediator linking obesity to insulin resistance and the pathogenesis of T2DM [78,79]. In obesity, expanding adipose tissue undergoes an immunologic shift. Hypertrophied adipocytes become stressed and release chemokines like MCP-1 that recruit immune cells into fat depots [78]. In particular, increased recruitment of monocytes that differentiate to inflammatory macrophages, together with a shift in resident macrophage polarization, results in a predominance of pro-inflammatory M1-like macrophages [78]. These M1 macrophages, along with other innate immune cells, secrete pro-inflammatory cytokines—notably TNF-α, IL-6, and IL-1β—which become elevated systemically in obese-associated, insulin-resistant states and in T2DM [78].

Such cytokines interfere with insulin signaling pathways in metabolic tissues. TNF-α, one of the first adipose-derived factors implicated in obesity-linked insulin resistance, is overexpressed in obese fat and can trigger serine phosphorylation of insulin receptor substrates (IRS) via JNK activation, thereby blunting downstream insulin signaling [78,79]. IL-6, another cytokine upregulated in obesity, can promote hepatic gluconeogenesis and dyslipidemia, further contributing to insulin resistance [79]. Collectively, this state of obesity-induced metaflammation disrupts normal insulin action in muscle, liver, and adipose tissue, creating a systemic insulin-resistant milieu that predispose to T2DM [78,79].

Importantly, obesity-related inflammation not only causes insulin resistance in peripheral tissues but also impairs pancreatic islet function directly. Pro-inflammatory cytokines (e.g., IL-1β and TNF-α) are often present in the islet microenvironment of T2DM, secreted by infiltrating islet macrophages or even by β-cells themselves under stress, inducing β-cell dysfunction and death [78]. IL-1β activates NF-κB and MAPK in β-cells, impairs glucose-stimulated insulin secretion, and triggers β-cells apoptosis [78]. Chronic hyperglycemia can provoke pancreatic β-cells to produce IL-1β, establishing a vicious autocrine loop that accelerates β-cell failure [78].

Consistent with these findings, T2DM patients typically show elevated circulating IL-1β along with IL-6, TNF-α and C-reactive protein, and the levels of these inflammatory markers correlate with insulin resistance and glycemic dysregulation [78]. This understanding has led to clinical trials targeting IL-1β. In a landmark study, treating T2DM patients with an IL-1 receptor antagonist (anakinra) for several months improved glycemic control and β-cell secretory function [80]. A meta-analysis of over 2,900 patients confirmed that IL-1 antagonism produces a modest but significant reduction in HbA1c, supporting a pathogenic role of IL-1β in T2DM [81]. However, these benefits are moderate, and IL-1 blockade has not yet become standard treatment for diabetes, indicating that targeting a single cytokine is insufficient to reverse the multi-factorial inflammatory process in T2DM [81].

The NLRP3 inflammasome links metabolic stress and inflammation in T2DM. This multiprotein complex, senses cellular-danger-signals and upon activation, cleaves pro-caspase-1 to caspase-1, which in turn matures the pro-inflammatory cytokines IL-1β and IL-18 [78]. In conditions of nutrient excess and obesity, a variety of DAMPs arising from metabolic stress can trigger NLRP3 assembly—for instance, elevated levels of ROS, excess free fatty acids (e.g., palmitate), ceramides, and even mitochondrial DNA released due to organelle dysfunction [13,78].

These triggers are plentiful in insulin-resistant obese tissues. Overnutrition leads to oxidative stress and lipotoxicity within cells, which signal through NLRP3 to initiate an inflammatory cascade. Numerous studies have implicated NLRP3 inflammasome activation in the chronic inflammation of T2DM. Overactivation of NLRP3 in metabolic tissues has been shown to interfere with insulin signaling and to promote the local release of IL-1β, exacerbating both insulin resistance and β-cell stress (Figure 1) [78]. In obese mouse models, genetic deletion of Nlrp3 confers protection against insulin resistance—*Nlrp3*-knockout mice on a high-fat diet exhibit improved insulin sensitivity and better glucose control than wild-type obese mice [82]. Conversely, treating cells with metabolic DAMPs that activate NLRP3 induces IL-1β production and impairs insulin signaling in those cells [78].

Clinically, there is evidence that inflammasome activity is higher in individuals with diabetes; for example, one study found that monocytes isolated from patients with T2DM produce excessive IL-1β due to heightened NLRP3 inflammasome activation [83]. This correlation between NLRP3 activation and poor glycemic control in humans underscores the translational relevance of inflammasome-driven inflammation in T2DM. As a result, NLRP3 has attracted interest as a potential therapeutic target to alleviate metabolic inflammation. Indeed, experimental NLRP3 inhibitors like MCC950 have been developed, and in preclinical studies, MCC950 effectively suppresses NLRP3 activation and downstream IL-1β release [84]. While such agents are not yet in clinical use, they represent a promising strategy to dampen the innate immune component of T2DM inflammation at its source.

Additionally, upstream regulators of inflammasome activity are being explored. For instance, activation of the NAD^+^-dependent deacetylase SIRT1 can indirectly inhibit NLRP3 and has been associated with reduced inflammation and improved metabolic function in animal models of diabetes [78]. These approaches illustrate how uncovering the role of NLRP3 in metaflammation is spurring new ideas to break the cycle of obesity-induced inflammation and metabolic disease. Several pharmacological agents targeting inflammation in T2DM have been evaluated in clinical and preclinical studies (Table 1), These agents act through diverse mechanism, such as IL-1β blockade, NF-κB pathway inhibition, and NLRP3 inflammasome suppression.

It is also now clear that the chronic inflammation in T2DM is not confined to macrophages alone, but involves a broad interplay of various immune cell types. Obesity-driven inflammation alters the balance of T lymphocytes in adipose and other tissues. Obesity-driven inflammation skews T cells toward pro-inflammatory subsets (such as Th1 and Th17 cells, and cytotoxic CD8^+ T cells) while diminishing anti-inflammatory or regulatory T cells [78]. These T cells produce cytokines (e.g., IFN-γ, IL-17) that can further activate macrophages and exacerbate insulin resistance in tissues. In parallel, B lymphocytes in obese adipose tissue can contribute to inflammation by secreting pro-inflammatory cytokines and even pathogenic antibodies that worsen metabolic dysfunction. Neutrophils, which infiltrate insulin-sensitive tissues in obesity, release neutrophil elastase and other mediators that have been linked to insulin resistance. Even mast cells and NK cells have been implicated in the obese inflammatory milieu.

Thus, both innate and adaptive immune responses are chronically activated in obesity, creating a persistent pro-inflammatory state that affects systemic metabolism [78]. Supporting this, experiments in mice have shown that deleting or suppressing T and B cells can alleviate adipose inflammation and improve insulin sensitivity, highlighting their contributory role in metabolic disease. Furthermore, gut microbiota dysbiosis has emerged as an upstream driver of chronic inflammation in obesity. A high-fat diet can alter the intestinal barrier and microbiome, leading to increased leakage of endotoxins such as lipopolysaccharide (LPS) from gram-negative bacteria into the circulation—a phenomenon termed metabolic endotoxemia [91]. Even a modest 2–3 fold rise in plasma LPS, well below levels seen in sepsis, is sufficient to trigger TLR4 receptors and chronically activate innate immune pathways [91].

Studies have linked these endotoxin increases to obesity-related insulin resistance and liver fat accumulation, suggesting that the gut-originating inflammation contributes to the systemic inflammatory burden in T2DM. In summary, obesity-associated inflammation is a multicellular, multi-tissue process: while M1-polarized adipose macrophages are central players, other leukocytes like Th1/Th17 cells, CD8^+^ T cells, B cells, neutrophils, etc., and even intestinal microbes participate in fueling the inflammatory milieu that promotes insulin resistance and β-cell stress [78].

A key concept in understanding this chronic inflammatory state is the bidirectional crosstalk between inflammation and cellular metabolism, particularly at the level of mitochondria. Inflammatory cytokines can directly perturb mitochondrial function in metabolic tissues. For instance, TNF-α and IL-1β are known to suppress mitochondrial respiration and biogenesis regulators (such as PGC-1α), and they can induce nitric oxide and other mediators that damage the mitochondrial electron transport chain [13]. This cytokine-induced mitochondrial dysfunction leads to reduced ATP production and accumulation of oxidative stress.

In turn, dysfunctional mitochondria emit signals that further amplify inflammation. Damaged or stressed mitochondria release ROS, oxidized lipids, and even mitochondrial DNA—all of which act as DAMPs that activate innate immune receptors like NLRP3 [13]. In adipose tissue macrophages exposed to excess fat, mitochondrial ROS and lipids (e.g., cardiolipin) have been shown to directly trigger inflammasome activation [78], linking mitochondrial stress to cytokine production. Thus, a vicious cycle is established whereby inflammation impairs mitochondrial health, and impaired mitochondria feedback to stimulate more inflammation.

In obesity and T2DM, evidence of this vicious cycle is apparent: patients demonstrate both inflammatory marker elevation and signs of mitochondrial abnormalities in muscle, liver, adipose, and β-cells. One contributing factor is impaired mitophagy—the reduced clearance of damaged mitochondria—which often accompanies overnutrition. When mitophagy is blunted (as has been observed in obese rodents and humans), dysfunctional mitochondria accumulate and continue to generate excessive ROS and inflammatory mitochondrial content, prolonging the activation of inflammasomes and other immune pathways [13]. Ultimately, the convergence of chronic inflammatory pathways and mitochondrial stressors drives the insulin resistance, β-cell failure, and tissue damage that characterize T2DM (Figure 1) [47,76,92,93]. This multifactorial pathogenesis helps explain the heterogeneity of T2DM and remains a focus of ongoing therapeutic exploration.

#### 2.1.4. Therapeutic Implications

Breaking this self-perpetuating loop is an emerging therapeutic goal in T2DM; however, translation to robust clinical benefit has been modest and heterogenous. Approaches that enhance mitochondrial quality control or reduce oxidative stress can have anti-inflammatory effects. For example, mitochondria-targeted antioxidants such as SS-31 (elamipretide) have been shown to reduce oxidative stress and inflammation in cellular and animal models of T2DM, in part by preserving mitochondrial function and activating sirtuins like SIRT1 [94]. Ex vivo treatment of leukocytes from patients with T2DM using the mitochondria targeted peptide SS-31 demonstrated reduced ROS and inflammatory cytokines along with increased SIRT1 activity [95], and animal studies have shown preserved mitochondrial integrity and improved tissue function [96]. While these data are encouraging, clinical validation in T2DM remains to be firmly established. Similarly, anti-inflammatory interventions such as IL-1β antagonism have produced only modest HbA1c reductions in clinical trials [86,97]. Likewise, interventions that stimulate autophagy/mitophagy may eliminate the ROS-producing mitochondria and thereby dampen inflammasome activity, but these approaches also require clinical confirmation.

In parallel, several pharmacological agents directly targeting mitochondrial and metabolic pathways are under clinical investigation (summarized in Table 2). For example, imeglimin enhances mitochondrial function and improves glycemic control; MSDC-0602K, a mitochondrial pyruvate carrier inhibitor, acts as an insulin sensitizer while sparing PPARγ activity; and BGP-15, an HSP72 co-inducer, alleviates oxidative and ER stress. Other agents, including HU6, Pegbelfermin, and SS-31 aim to restore mitochondrial quality, improve lipid oxidation, and dampen inflammation. These examples underscore the growing therapeutic focus on mitochondrial biology as a means to counteract the inflammatory-metabolic cycle in T2DM.

Exercise is one well-recognized modality that can improve mitochondrial function and concurrently lower chronic inflammation. Regular physical exercise has been found to shift the balance of cytokines, increasing anti-inflammatory mediators (like IL-10 and IL-1ra) and decreasing levels of CRP, TNF-α, and IL-6 in patients with T2DM [98]. These changes are accompanied by improved insulin sensitivity. In fact, weight loss through diet and exercise is known to reduce adipose macrophage content and inflammatory gene expression, underlining how lifestyle can modulate the inflammation–metabolism axis.

Other potential strategies include activation of AMP-activated kinase (AMPK) and certain polyphenols (e.g., resveratrol, a SIRT1 activator), which have demonstrated anti-inflammatory and insulin-sensitizing effects in preclinical studies of obesity—partly by enhancing mitochondrial efficiency and antioxidant defenses [78]. While many of these approaches are still under investigation, they align with the concept that targeting the inflammatory–mitochondrial feedback loop could yield metabolic benefits. In addition to inflammation-targeted therapies, several agents designated to restore mitochondrial function and improve metabolic flexibility are under development for T2DM (Table 2). These include mitochondrial-targeted antioxidants, MPC inhibitors, HSP co-inducers, and FGF21 analogs.

In summary, chronic inflammation is a fundamental feature and pathogenic driver of T2DM, especially in the context of obesity. Recognizing the central role of inflammation in T2DM has opened new avenues for treatment—from anti-cytokine biologics to small-molecule inflammasome inhibitors and lifestyle interventions—aimed at restoring immunometabolic balance. Notably, clinical trials neutralizing IL-1β or using broad anti-inflammatory drugs have shown improvements in glycemia and inflammatory markers, albeit with only modest efficacy as monotherapy [80,81]. This suggests that an individualized, multi-targeted approach (combining metabolic therapy with inflammation reduction and mitochondrial support) may be necessary to meaningfully interrupt the disease progression. Ongoing work could lead to therapies that maintain insulin sensitivity and β-cell function. The convergence of metabolic and immune pathways underscores that T2DM is not purely endocrine, but also immune-mediated [78,79].

**Table 2:** Drug Targeting Mitochondrial/Metabolic Pathways in T2DM.

Drug	Mechanism & Molecular Target	Developer	ClinicalTrials.gov Identifier	Phase	References
**Imeglimin**	Oral *Glimin* class; enhances mitochondrial function	Poxel & Sumitomo Dainippon; approved by Japan’s PMDA in 2021 as Twymeeg^®^. Approval was based on Phase 3 trials in Japan. In U.S., only Phase II trials has been completed.	N/A	**2 (FDA)**	[99,100]
**MSDC-0602K**	*Azemiglita zone*; MPC inhibitor; PPARγ-sparing insulin sensitizer targeting mitochondrial pyruvate carrier (mTOT)	Cirius Therapeutics (Metabolic Solutions)	NCT02784444 (EMMINECE), NCT03970031 (planned)	**3**	[101]
**BGP-15**	HSP modulator; orally active co-inducer of heat shock proteins (HSP72); reduces oxidative and ER stress	N-Gene/Mitochon (HU)	NCT01069965	**2a**	[102]
**HU6**	Mitochondrial uncoupler	Rivus Pharmaceuticals (Phase 2b in obesity+T2DM ongoing)	NCT04874233	**2b**	[103]
**Pegbelfermin**	FGF21 analog; enhances lipid oxidation, weight loss, and insulin-independent glucose uptake	Bristol Myers Squibb (BMS-986036)	NCT02071509	**2a**	[104]
**SS-31 (Elamipretide)**	Selectively binds to Cardiolipin in inner mitochondrial membrane, stabilizing mitochondrial structure and function	Stealth Bio Therapeutics (developing for Barth syndrome)	N/A (Preclinical Studies Only)	**N/A**	[94]

### 2.2. Metabolic Dysfunction-Associated Steatotic Liver Disease (MASLD)

#### 2.2.1. Overview of MASLD

Metabolic dysfunction-associated steatotic liver disease (MASLD) represents the contemporary nomenclature for what was previously termed non-alcoholic fatty liver disease (NAFLD). This diagnosis falls within the broader category of steatotic liver disease (SLD) and is characterized by hepatic fat accumulation in conjunction with one or more cardiometabolic risk factors, including obesity, hypertension, diabetes, dyslipidemia, or insulin resistance [105]. If metabolic risk factors present alongside moderate or higher alcohol intake, the diagnosis of Metabolic dysfunction-associated alcohol-related liver disease (MetALD), a condition considered distinct from MASLD [105]. The global prevalence of MASLD is estimated at approximately 38% among adults, with incidence rates continuing to escalate [106]. The condition is particularly prevalent in individuals with obesity (body mass index ≥ 30) and may progress from simple steatosis to metabolic dysfunction-associated steatohepatitis (MASH), cirrhosis, and hepatocellular carcinoma (HCC) [107,108]. Overnutrition and obesity are recognized as principal factors that substantially elevate the risk of MASLD by inducing mitochondrial dysfunction and triggering inflammatory pathways [109,110].

#### 2.2.2. Mitochondrial Dysfunction in MASLD Progression

**Figure 2:**
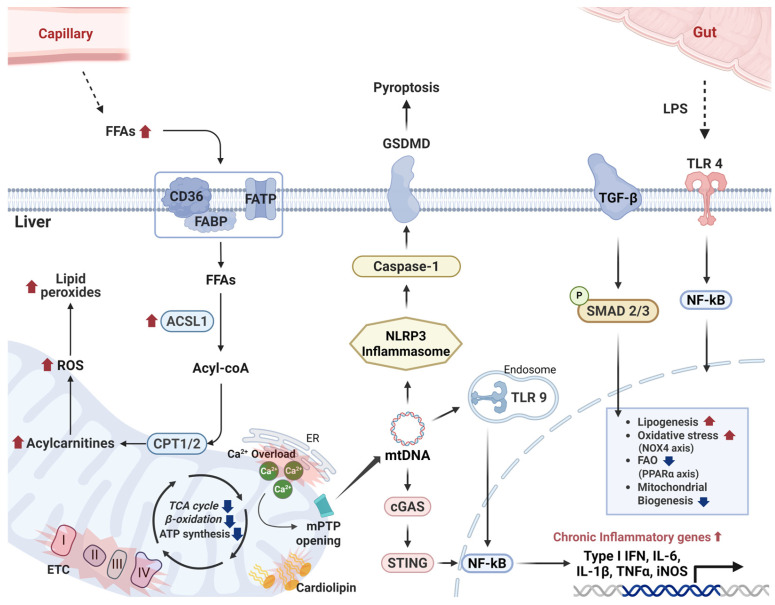
Integrated model of obesity-driven FFA overload reprogramming hepatocellular metabolic–immune axes. Chronic elevation of FFAs in obesity hyperactivates the hepatocellular influx–transport–activation–oxidation cascade, creating a β-oxidation bottleneck with increased mtROS, mPTP opening, and mtDNA release. In parallel, LPS–TLR4 signaling from the gut–liver axis and TGF-β–SMAD2/3–mediated nuclear reprogramming converge to amplify NF-κB activation, pro-inflammatory cytokine output (IL-1β, IL-6, TNFα, Type I IFN), and pyroptosis. (1) FFA influx and activation (plasma → hepatocyte). Elevated FFAs enter hepatocytes via CD36, FABP1, and FATP5/SLC27A5. Cytosolic fatty acids are converted to acyl-CoA by ACSL1 for mitochondrial import. (2) Mitochondrial handling. Long-chain acyl groups are transferred into mitochondria through the carnitine shuttle (CPT1/2). Under overload, β-oxidation and ATP synthesis decline, while acylcarnitines accumulate as a marker of flux limitation. ROS and lipid peroxides rise; cardiolipin alteration together with Ca^2+^ overload promotes mPTP opening. (3) mtDNA release and innate sensing. Damaged mitochondria release mtDNA, which activates cytosolic cGAS, driving the STING → NF-κB/Type I IFN axis. In parallel, the NLRP3 inflammasome is engaged, leading to pro-caspase-1 cleavage → caspase-1, GSDMD pore formation, and pyroptosis. (4) Gut–liver axis. Intestinal LPS engages hepatocellular TLR4 to reinforce NF-κB signaling. TLR9, localized to the endosome, recognizes CpG-DNA/mtDNA (shown as a separate vesicular bubble). (5) Nuclear reprogramming (TGF-β–SMAD2/3). Upon TGF-β stimulation, phosphorylated SMAD2/3 translocate to the nucleus and reshape metabolic, redox, and mitochondrial programs. Directionality (right panel): lipogenesis ↑; NOX4 ↑ (increasing NOX4-dependent ROS tone); FAO genes ↓—notably the PPARα-driven FAO module (e.g., CPT1A, ACOX1)—and mitochondrial biogenesis ↓—attenuation of the PGC-1–NRF–TFAM module. Graphic conventions. Solid arrows, pathway flow/activation; bold ↑/↓, up/down regulation; red labels, stress/accumulation; dashed arrows, derived inputs (e.g., FFAs, LPS) (Created in https://BioRender.com).

In obesity, elevated FFAs chronically overload hepatocyte lipid handling systems through transport and activation proteins such as CD36, FATP5, FABP1, and ACSL1/5 [111,112,113,114,115,116]. CD36 overexpression, stimulated by obesity, hyperglycemia, and O-GlcNAcylation, accelerates hepatic lipid accumulation and inflammation [111]. Conversely, FATP5 deficiency can worsen fibrosis by activating hepatic stellate cells via unconjugated bile acid accumulation [113]. FABP1 directs fatty acid flux between β-oxidation and esterification, and its circulating levels correlate with disease severity [117]. ACSL1 promotes β-oxidation, while ACSL5 is stabilized through USP29-mediated deubiquitination or the SIRT6 pathway to enhance β-oxidation Furthermore, the anti-apoptotic protein MCL-1 interacts with ACSL1 to potentiate long-chain fatty acid oxidation [118]. Collectively, this “influx–transport–activation–oxidation” axis is hyperactivated in obesity, leading to mitochondrial overload and hepatocellular metabolic stress (Figure 2) [111,112,119].

Excessive FFA influx saturates the carnitine shuttle system (CPT1A, CACT, CPT2) and PPARα signaling [120]. As a compensatory mechanism, peroxisomal β-oxidation is induced via ACOX1 but generates excess H_2_O_2_, contributing to oxidative stress and insulin resistance [119]. A bottleneck at the electron transfer flavoprotein (ETF)–electron transfer flavoprotein dehydrogenase (ETFDH) step increases reductive pressure, causing electron leakage and mtROS overproduction [38,121]. Consequently, C14–C16 acylcarnitines accumulate in hepatic tissue and circulation, representing a consistent metabolic hallmark of MASLD across clinical cohorts [112,122]. The resulting incomplete oxidation places further stress on the ETC [38].

Electron leakage at Complexes I and III amplifies ROS generation, destabilizes mitochondrial membrane potential (Δψm), reduces ATP synthesis, and induces mtDNA damage [38,123]. OXPHOS impairment is not limited to energy deficits but also includes lipid peroxidation, protein oxidation, DNA damage, and activation of cellular stress responses and programmed cell death pathways [123,124]. Declines in Δψm are recognized as early indicators of lipotoxicity and have been consistently observed in cell culture, animal models, and human livers with advanced fibrosis [125,126,127,128]. Moreover, clinical ^31^P-MRS studies demonstrate persistent reductions in hepatic ATP synthesis and impaired recovery after nutrient challenge, with efficiency further diminished in advanced fibrosis [128,129]. Despite elevated AMP/ATP ratios, AMPK activity is paradoxically suppressed in obesity and overnutrition, preventing compensatory responses [130,131].

ROS accumulation correlates with lipid peroxidation products including 4-hydroxynonenal and malondialdehyde [124,132]. Palmitate exposure reduces peroxiredoxin-1 activity, weakening antioxidant defenses and exacerbating MASH [127]. Excessive ROS also drive release of mtDAMPs, which activate the NLRP3 inflammasome and NF-κB signaling in Kupffer cells and macrophages [133,134].

Mitochondrial membrane remodeling further contributes to dysfunction. Cardiolipin loss destabilizes ETC supercomplexes (respirasomes), enhancing electron leakage from Complex III and Complex II flavin sites [123,125,135,136]. This mechanism, combined with decreased mtDNA transcription, protein folding stress, and redox imbalance, aggravates mitochondrial injury [11,123,124].

Another crucial axis involves mitochondria-associated membranes (MAMs), which regulate metabolic homeostasis and stress responses. HFD disrupts the IP3R–GRP75–VDAC complex and the MICU1/mitochondrial calcium uniporter (MCU) calcium uptake axis, impairing calcium signaling and lipid dynamics [137,138,139,140]. Seipin, localized at mitochondria–ER contacts, modulates lipid droplet biogenesis and hepatic lipogenesis, linking MAMs to lipid metabolic abnormalities [137]. Disruption of MAMs and mitochondrial calcium homeostasis promotes mPTP opening, with overactivation of cyclophilin D amplifying hepatocyte injury and inflammation [141,142,143,144,145]. Inhibition of the MCU attenuates fibrosis and inflammation in MASH models [146,147]. ER stress converges with mitochondrial calcium overload, producing ROS and activating the NLRP3 inflammasome (Figure 2) [148]. VDAC1 oligomerization has also been proposed as a mechanistic contributor to inflammasome activation [149].

As ROS and lipid overload persist, mtDNA becomes oxidized and fragmented, increasing membrane permeability through mPTP opening and BAX/BAK pore formation [38,150,151]. Cytosolic and extracellular mtDNA engage pattern recognition receptors, including endolysosomal TLR9 and cytosolic cGAS–STING [152]. Activation of these sensors triggers TBK1–IRF3–NF-κB signaling, fueling hepatic inflammation and fibrogenesis [38,153,154]. Elevated plasma mtDNA and TLR9 activation have been documented in MASH patients and animal models [152]. TLR9 inhibition reduces inflammation and tissue injury, while STING deficiency in Kupffer cells dampens NF-κB activation and cytokine expression [153,154]. Systemic STING inhibition similarly alleviates steatosis, inflammation, and fibrosis [154].

Recent studies have highlighted additional regulatory nodes. The FoxO1–YAP–Notch1 axis in hepatic macrophages reprograms cGAS–STING-mediated innate immunity [155]. CMPK2, an enzyme involved in mtDNA synthesis, is selectively upregulated in hepatocytes during MASH; its inhibition suppresses NLRP3 inflammasome activation and pyroptosis [156]. Downregulation of mitochondrial transcription factor A (TFAM) destabilizes mtDNA and disrupts mitochondrial function, whereas TFAM restoration via the PGC-1α–NRF2 pathway enhances mitochondrial homeostasis and reduces injury [157].

Pyroptosis emerges as a central form of inflammatory cell death. Elevated ROS, Δψm collapse, and VDAC1 oligomerization promote K^+^ efflux and mtDNA release, activating the NLRP3–caspase-1–GSDMD pathway [110,149,158,159,160,161,162]. Gasdermin D pore formation leads to acute secretion of IL-1β and IL-18 and release of DAMPs, further amplifying inflammation [163,164]. BRD4 inhibition reduces VDAC1 oligomerization and prevents pyroptosis while stabilizing Δψm [149]. PINK1/Parkin-mediated mitophagy attenuates inflammasome priming, lowering pyroptotic susceptibility [161,165,166]. Hepatocyte-specific NR5A2 deletion activates the ROS–NF-κB–NLRP3–caspase-1–GSDMD axis via ALDH1B1 suppression, whereas ALDH1B1 restoration counteracts pyroptosis and inflammation [167]. Additionally, necroptosis, mediated by RIPK3 and MLKL, as well as ferroptosis, contribute to the pathogenesis of MASLD, with ferroptosis identified as a particularly actionable deleterious factor [168,169,170]. The concurrent activation of these programmed cell death pathways establishes a deleterious feedback loop via the re-release of mtDAMPs, thereby accelerating the progression from inflammation to fibrosis (Figure 2) [110,158,159,168].

In summary, obesity-driven lipid overload precipitates a cascade of mitochondrial perturbations—including incomplete β-oxidation, ETC dysfunction, MAM–Ca^2+^ imbalance, mtDAMP release, and programmed cell death—that converge to amplify chronic inflammation and fibrosis, thereby constituting a central axis in the progression of MASLD. Thus, mitochondrial dysfunction not only disrupts cellular bioenergetics but also generates danger signals that prime and amplify hepatic inflammation, setting the stage for the immunopathological cascades discussed in Section 2.2.3.

#### 2.2.3. Hepatic Inflammation in MASLD

Building upon these mitochondrial signals, the progression to MASH is orchestrated by the activation and crosstalk between liver-resident Kupffer cells and recruited monocytes/macrophages [171,172,173]. As simple steatosis advances, DAMPs from lipotoxic hepatocytes and pathogen-associated molecular patterns entering via a leaky gut activate Kupffer cells, escalating IL-1β/TNF-α production and fueling hepatocellular injury and fibrogenic signaling [172,174,175,176].

Among the molecular regulators shaping these macrophage responses, hypoxia-inducible factor-2α (HIF-2α) has drawn increasing attention [172]. As an oxygen-sensing transcription factor involved in immune and metabolic regulation, HIF-2α has been recognized as a pivotal modulator of hepatic macrophage function, notably influencing Kupffer cell survival, efferocytosis, and inflammatory activity in MASH models. Hyperactivated HIF-2α reshapes Kupffer cell survival and metabolism, amplifying IL-1β and TNF-α, while its pro-apoptotic effect promotes compensatory recruitment of monocyte-derived macrophages enriched in inflammatory gene expression [172,175,177,178]. In a choline-deficient L-amino acid-defined HFD model, however, the ceramide-derived metabolite sphingosine d18:1 [So(d18:1)] suppresses macrophage HIF-2α, and macrophage-specific restoration of HIF-2α reduces NLRP3 inflammasome signaling, mitigating inflammation and fibrosis [173,179]. Together, these findings suggest that while excessive HIF-2α drives inflammation, a baseline level is required to restrain inflammasome overactivation.

Kupffer cells and infiltrating macrophages secrete CCL2, recruiting CCR2^+^ monocytes that, together with resident cells, release additional TNF-α and IL-1β. These cytokines activate hepatic stellate cells (HSCs) via TGF-β, initiating collagen deposition and linking inflammation to fibrosis [174,180,181,182]. Adaptive and innate lymphocytes further reinforce this milieu: CD8^+^ T cells and NK cells accumulate and secrete IFN-γ, while neutrophils form NETs enriched in MPO and histones, directly injuring hepatocytes and mitochondria [183,184,185,186]. NET-driven tissue damage enhances reactive oxygen species and lipid peroxidation, establishing a feed-forward loop of immune recruitment and persistent inflammation [186,187]. Collectively, the coordinated network centered on Kupffer cells and monocyte/macrophage populations underlies MASLD progression from steatosis to MASH characterized by pronounced inflammatory injury [171,174,188].

As inflammatory pathways intensify, cytokines such as TNF-α and IL-1β impair hepatocyte mitochondrial biogenesis and quality control [189,190]. Persistent NF-κB and JNK activation downregulates PGC-1α and TFAM, lowering mitochondrial content and functional capacity [189,191]. PGC-1α expression is markedly reduced in advanced MASH and inversely correlates with inflammation and fibrosis [191,192].

Reduced TFAM destabilizes mtDNA and suppresses ETC protein expression, further limiting mitochondrial respiration [193]. Consequently, ATP production declines while ROS generation rises in LPS-stimulated Kupffer cells, α-ketoglutarate attenuates PKCε/MAPK/NF-κB signaling, lowers TNF-α, IL-6, and ROS, and restores OXPHOS [127,194,195]. Hepatocyte-specific IL-1R1 inhibition reduces inflammatory gene expression and fibrotic programs, demonstrating that IL-1β–NF-κB signaling depresses mitochondrial respiration and membrane potential even under physiological conditions [196,197]. Chronic inflammation further aggravates mitochondrial quality control by impairing clearance of damaged mitochondria [198].

Cytokine signaling activates mTOR and inhibits autophagic flux, particularly selective mitophagy [199,200]. It is known that MASLD/MASH models show p62 accumulation and reduced LC3-I to LC3-II conversion, consistent with inflammation-induced autophagy blockade [201]. The accumulation of dysfunctional mitochondria exacerbates stress through electron-leak–driven ROS and ATP depletion [202,203]. mtDNA and protein fragments released from damaged mitochondria activate NLRP3 and TLR9, amplifying inflammatory signaling [204]. Conversely, pharmacologic activation of autophagy/mitophagy facilitates clearance of damaged mitochondria and reduces liver inflammation and injury in multiple models [194,205].

Microbial products derived from dysbiosis contribute further to mitochondrial suppression through the gut–liver axis. For example, LPS translocates via the portal vein due to dysbiosis and barrier disruption, engaging TLR4 on Kupffer cells to trigger innate immune activation [206,207,208]. TLR4 signaling induces NF-κB–dependent cytokine release and iNOS, with the resulting nitric oxide impairing ETC function and oxidative phosphorylation in hepatocytes and immune cells [209,210,211]. Under LPS stimulation, innate immune cells activate HIF-1α and shift toward glycolysis, a Warburg-like reprogramming that reduces hepatic energetic efficiency [209,212]. Interventions such as the antimicrobial peptide nisin lower portal LPS, hepatic oxidative stress, and steatosis in dysbiosis-driven inflammation [213,214]. Modulation of LPS-binding protein alters hepatocyte lipid metabolism and oxidative stress, linking PAMP signaling directly to hepatic metabolism [215,216]. Chronic low-grade endotoxemia with elevated circulating LPS sustains TLR4 activation in MASLD, driving persistent inflammation and metabolic dysfunction [216,217].

These microbial cues intersect with mitochondrial stress via DNA-sensing pathways. Microbial or mitochondrial DNA fragments activate immune sensors through TLR9 and cGAS–STING, amplifying inflammation [204,218]. TLR9 induces cytokines and interferons through MyD88–NF-κB/IRF, while cGAS–STING activates TBK1–IRF3 to elicit similar programs [218,219]. Hepatocyte-derived mtDNA is sensed by macrophage TLR9, provoking interferon-mediated injury [220]. Genetic or pharmacologic inhibition of cGAS–STING lowers ALT/AST, cell death, and inflammation in ischemia–reperfusion and MASH models, while macrophage depletion abrogates pathway activation [219,221]. In sum, PAMP/DAMP recognition via the gut–liver axis suppresses mitochondrial respiration, enforces metabolic reprogramming, and sustains chronic hepatic inflammation (Figure 2) [208,217,221].

As fibrosis develops, activated HSCs impose additional metabolic suppression on hepatocytes. TGF-β secreted by HSCs reprograms hepatocytes via Smad2/3 by upregulating SREBP-1c to drive lipogenesis while suppressing PPARα and its FAO targets (CPT1A, ACOX1), thereby reducing β-oxidation [222,223,224,225,226,227].

TGF-β also downregulates PGC-1α and TFAM, impairing mitochondrial biogenesis and mtDNA replication [224]. Consequently, hepatocytes accumulate triglycerides, produce less energy, and generate more ROS, creating a deleterious metabolic environment [226,228]. In parallel, TGF-β increases NOX4 activity in hepatocytes and HSCs, amplifying H_2_O_2_ production and profibrogenic signaling [229,230]. This rewiring sustains lipotoxic stress and reinforces a self-perpetuating TGF-β–fibrosis cycle (Figure 2) [222,223,226,228,229].

Attenuating TGF-β/Smad signaling in animal models ameliorates steatosis, inflammation, and fibrosis, with partial restoration of FAO-related genes, whereas pathway hyperactivation accelerates lipid loading and injury [222,223,224,231]. These findings support a direct role for TGF-β–mediated metabolic suppression in MASLD pathogenesis. Conversely, PPARα activation enhances FAO and ketogenesis, lowers inflammatory mediators, and helps restore hepatic metabolism PPAR agonists (e.g., pemafibrate, lanifibranor) and TGF-β inhibitors reduce hepatic lipid accumulation, inflammation, and fibrosis markers in preclinical and clinical settings, underscoring the therapeutic potential of targeting the metabolism–fibrosis axis in MASLD [222,232,233,234].

#### 2.2.4. Therapeutic Implications

Obesity-driven MASLD is now recognized by recent guidelines and reviews as a disease state propelled by a self-reinforcing loop between hepatic mitochondrial dysfunction and innate–adaptive immune inflammation [235,236]. Therapeutically, this biology maps onto two complementary axes: (i) systemic–metabolic therapies that restore whole-body energy balance and lower hepatic lipotoxic substrate pressure (e.g., GLP-1–based agents), and (ii) metabolic-targeted hepatocellular therapies that directly reprogram lipid and mitochondrial metabolism (e.g., THR-β, PPAR, FGF21) [235,236]. On the regulatory front, resmetirom (THR-β agonist) received accelerated approval in 2024 for non-cirrhotic MASH with F2–F3 fibrosis, establishing the first FDA-authorized therapy for this population under the accelerated-approval pathway [237]. In August 2025, semaglutide (Wegovy, GLP-1 RA) likewise obtained accelerated approval for MASH with moderate–advanced fibrosis on the basis of 72-week histologic surrogate endpoints; continued approval may depend on verification and description of clinical benefit in confirmatory trials, consistent with the accelerated-approval framework [238,239].

Among agents that simultaneously address mitochondrial dysfunction + inflammation, lanifibranor (pan-PPAR) upregulates β-oxidation and PGC-1α through PPAR-α/δ while repressing inflammatory transcription via PPAR-γ; in the phase-2b NATIVE RCT it achieved both MASH resolution and ≥1-stage fibrosis improvement [240]. Efruxifermin (EFX, an FGF21 analog) achieved, in non-cirrhotic F2–F3 MASH patients in the HARMONY phase-2b trial, ≥1-stage fibrosis improvement and MASH resolution at 24 weeks—with concurrent achievement rates also superior to placebo—findings that align with the FGF21 → adiponectin → AMPK/SIRT1 → PGC-1α axis. In contrast, in compensated cirrhosis (F4) in the SYMMETRY phase-2b trial, the 36-week primary endpoint was not met; however, exploratory 96-week analyses showed signals of fibrosis improvement and cirrhosis reversal, suggesting a potential time-dependent benefit with longer treatment [240,241]. Pegozafermin (FGF21 analog) similarly produced MASH resolution and fibrosis improvement in a phase-2b study, reinforcing the same FGF21 pathway mechanism for mitochondrial functional recovery [242,243]. Survodutide (GLP-1/Glucagon dual) improved MASH without fibrosis worsening in phase-2, and human studies show glucagon-receptor activation increases hepatic mitochondrial oxidation and pyruvate carboxylase flux, providing direct metabolic evidence for this class [244,245]. In parallel, tirzepatide (GIP/GLP-1 dual) achieved MASH resolution in phase-2b and strengthens the causal chain linking weight loss, hepatic fat reduction, and improved insulin resistance to lower mitochondrial stress and downstream histologic improvement (Table 3) [246].

In summary, obesity-driven MASLD is increasingly understood as a disease sustained by the interplay between mitochondrial dysfunction and chronic immune activation. Recent accelerated approvals of resmetirom and semaglutide indicate that both hepatocyte-targeted mitochondrial reprogramming and systemic metabolic correction can deliver histologic benefits, establishing proof-of-concept for disease modification under this regulatory pathway. Emerging therapies—including pan-PPAR agonists, FGF21 analogs, and incretin dual agonists—extend this paradigm by enhancing β-oxidation, restoring mitochondrial biogenesis, and reducing inflammatory signaling. Notably, trials of lanifibranor, efruxifermin, and pegozafermin have shown improvements in both steatohepatitis resolution and fibrosis stage, outcomes previously elusive in MASLD drug development. Still, efficacy varies across patient populations—particularly those with advanced fibrosis—and long-term durability remains uncertain, suggesting that individualized, multi-targeted strategies (combining weight reduction, inflammation control, and mitochondrial support) may be required to meaningfully alter disease trajectories. Ongoing work aims to refine patient stratification with biomarkers (e.g., circulating mtDNA, acylcarnitines) and to integrate metabolic- and immune-directed therapies. Genotype (e.g., PNPLA3) currently functions as a research/adjunctive risk-stratification tool rather than a routine determinant of treatment selection, with NIT-based stratification remaining the primary framework in contemporary guidelines [246]. Together, these advances indicate that MASLD is no longer managed solely by lifestyle intervention but is entering an era of mechanism-based, disease-modifying treatment with real potential to slow or reverse progression.

## 3. Conclusions

T2DM and MASLD both closely linked to obesity, are complex disorders driven by intricate interactions between chronic low-grade inflammation and mitochondrial dysfunction. In these conditions, excessive nutrient intake and lipid accumulation impose a burden on mitochondria, impairing ATP production efficiency and generating the excessive stress signals, such as ROS.

Mitochondrial stress result in the release of mtDAMPs, which activate key inflammatory signaling pathways, including the NLRP3 inflammasome, NF-κB, and JNK. The subsequent activation of these inflammatory cascades contributes to systemic insulin resistance and pancreatic β-cell injury, thereby accelerating the progression of both T2DM and MASLD. Concurrently, pro- inflammatory cytokines further impair mitochondrial function, creating a deleterious feedback loop that exacerbates tissue damage and disease severity.

Consequently, effective management of T2DM and MASLD requires a comprehensive strategy targeting both metabolic dysfunction and inflammation rather than focusing narrowly on glycemic control or hepatic steatosis alone. Therapeutic interventions that concurrently target the interplay between mitochondrial dysfunction and inflammation are likely to play a pivotal role halting or retarding disease progression and improving clinical outcomes.

## Figures and Tables

**Table 1:** Drug Targeting Inflammation in T2DM.

Drug	Mechanism & Molecular Target	Developer	ClinicalTrials.gov Identifier	Phase	References
**Anakinra**	Recombinant IL-1 receptor antagonist; blocks IL-1α/β signaling	Swedish Orphan Biovitrum	NCT00303394	**2**	[80]
**Canakinumab**	Human monoclonal antibody against IL-1β; reduces systemic inflammation	Novartis	NCT01327846 (CANTOS trial)	**3 CVOT**	[85]
**LY2189102**	Neutralizing IL-1β; inhibits IL-1β mediated inflammatory signaling	Eli Lilly	NCT00711556	**2**	[86]
**Salsalate**	Non-acetylated salicylate; inhibits IKKβ/NF-κB activation	Generic; studied in T2DM	NCT00799643 (TINSAL trials by NIH)	**2/3**	[87]
**Colchicine**	Anti-inflammatory alkaloid; disrupts microtubules, inhibiting NLRP3 inflammasome activation	Generic; repurposed for T2DM inflammation (on going trials)	NCT04181996 (CADENCE trial)	**3**	[88,89]
**Dorzagliatin**	First-in-class glucokinase allosteric activator; restores glucose sensing in β-cells & liver	Approved by China’s NMPA in 2022 as HuaTangNing^®^ for T2DM. Completed Phase 3 trials in China. No approval in the U.S.	NCT03173391 & NCT03141073 (DAWN)	**N/A**	[90]
**MCC950**	Direct NLRP3 inflammasome inhibitor	Pfizer (CP-456773/CRID3); halted due to drug-induced hepatotoxicity	No T2DM clinical trial (Pre-clinical ONLY)	**N/A**	[84]

**Table 3:** Key Investigational Drugs Targeting Metabolic–Inflammatory Axes in MASLD.

Drug	Target	Developer	ClinicalTrials.gov Identifier	Phase	References
Lanifibranor	pan-PPAR (α/δ/γ) agonist	Inventiva	NCT04849728	Phase 3 (ongoing)	[240]
Efruxifermin (EFX)	FGF21 analogue (FGFR1c/2c/3c agonism)	Akero Therapeutics	NCT06215716	Phase 3 (ongoing)	[241]
Pegozafermin	FGF21 analogue	89bio	NCT06318169	Phase 3 (ongoing)	[242,243]
Survodutide (BI-456906)	Dual GLP-1/Glucagon receptor agonist	Boehringer Ingelheim · Zealand Pharma	NCT06632444; NCT06632457	Phase 3 (ongoing)	[244,245]

## Data Availability

No new data were created or analyzed in this study. Data sharing is not applicable to this article.

## Abbreviations

The following abbreviations are used in this manuscript:

ACOX1	Acyl-CoA oxidase 1
ACSL1	Acyl-CoA synthetase long-chain family member 1
ACSL5	Acyl-CoA synthetase long-chain family member 5
AMPK	AMP-activated protein kinase
BAT	Brown adipose tissue
BAX	BCL2-associated X protein
BGP-15	Hydroximic acid derivative BGP-15
BMI	Body mass index
CACT	Carnitine-acylcarnitine translocase
CARD	Caspase recruitment domain
CCL2	C-C motif chemokine ligand 2
cf-mtDNA	Cell-free mitochondrial DNA
CMPK2	Cytidine/uridine monophosphate kinase 2
CPT1A	Carnitine palmitoyltransferase 1A
CPT2	Carnitine palmitoyltransferase 2
CRID3	Cytokine release inhibitory drug 3
CRP	C-reactive protein
CS	Citrate synthase
CVOT	Cardiovascular outcome trial
DAG	Diacylglycerol
DAMPs	Damage-associated molecular patterns
Δψm	Mitochondrial membrane potential
ETC	Electron transport chain
ETF	Electron transfer flavoprotein
ETFDH	Electron transfer flavoprotein dehydrogenase
FABP1	Fatty acid-binding protein 1
FAO	Fatty acid β-oxidation
FFAs	Free fatty acids
FGF21	Fibroblast growth factor 21
GAS	Gastrocnemius
GCN2	General control nonderepressible 2
GSIS	Glucose-stimulated insulin secretion
GSDMD	Gasdermin D
HCC	Hepatocellular carcinoma
HFD	High-fat diet
HIF	Hypoxia-inducible factor
HOMA-β	Homeostatic model assessment of β-cell function
HSP	Heat shock protein
IL	Interleukin
IP3R	Inositol 1,4,5-trisphosphate receptor
IRS	Insulin receptor substrate
JAK	Janus kinase
JNK	c-Jun N-terminal kinase
LPS	Lipopolysaccharide
MAMs	Mitochondria-associated membranes
MASLD	Metabolic dysfunction-associated steatotic liver disease
MASH	Metabolic dysfunction-associated steatohepatitis
MCC950	NLRP3 inflammasome inhibitor MCC950
MCL-1	Myeloid cell leukemia 1
MetALD	Metabolic dysfunction-associated alcohol-related liver disease
MLKL	Mixed lineage kinase domain-like protein
MPC	Mitochondrial pyruvate carrier
mPTP	Mitochondrial permeability transition pore
mtDNA	Mitochondrial DNA
mtDAMPs	Mitochondrial damage-associated molecular patterns
mTOT	Mitochondrial target of thiazolidinediones
NAFLD	Non-alcoholic fatty liver disease
NF-κB	Nuclear factor kappa-light-chain-enhancer of activated B cells
NK	Natural killer
NLRP3	NOD-, LRR- and pyrin domain-containing protein 3
NOX4	NADPH oxidase 4
OXPHOS	Oxidative phosphorylation
PAMPs	Pathogen-associated molecular patterns
PAX4	Paired box gene 4
PGC-1α	Peroxisome proliferator-activated receptor gamma coactivator 1-alpha
PKC	Protein kinase C
PPARα	Peroxisome proliferator-activated receptor alpha
PRRs	Pattern recognition receptors
RIPK3	Receptor-interacting serine/threonine-protein kinase 3
ROS	Reactive oxygen species
SIRT1	Sirtuin 1
SLD	Steatotic liver disease
SREBP-1c	Sterol regulatory element-binding protein 1c
STING	Stimulator of interferon genes
T2DM	Type 2 diabetes mellitus
TBK1	TANK-binding kinase 1
TFAM	Mitochondrial transcription factor A
TGF-β	Transforming growth factor beta
TLR4	Toll-like receptor 4
TLR9	Toll-like receptor 9
TNF-α	Tumor necrosis factor alpha
UCP1	Uncoupling protein 1
USP29	Ubiquitin-specific protease 29
VDAC	Voltage-dependent anion channel
